# Food Addiction Symptoms in Adults with Alcohol Use Disorder

**DOI:** 10.64898/2026.08.11.26360166

**Published:** 2026-08-12

**Authors:** Jennifer J. Barb, Li Yang, Jenny Yarmovsky, Melanie Schwandt, Vijay Ramchandani, Nancy Diazgranados, Ashley N. Gearhardt, Lorenzo Leggio

## Abstract

**Importance:**

Food addiction (FA) has been proposed as a phenotype sharing features with substance use disorders. Despite increasing recognition of food addiction as a behavioral phenotype with features overlapping substance use disorders, little is known about its prevalence or clinical significance among individuals with alcohol use disorder (AUD).

**Objective:**

To examine the prevalence of FA and to evaluate demographic, psychological, and alcohol related correlates in individuals with AUD.

**Design, Setting, and Participants:**

This cross-sectional analysis included 743 adults with AUD who were either treatment-seeking (Tx) (n = 534) for AUD and were enrolled in an inpatient program at the National Institutes of Health Clinical Center or non–treatment-seeking (non-Tx) (n = 209).

**Main Outcomes and Measures:**

FA symptoms were assessed using the Yale Food Addiction Scale, with ≥2 symptoms categorized as FA in this report. Multivariable logistic regression models adjusted for age, education, and income were conducted separately within each cohort.

**Results:**

Among 743 adults with AUD, 238 (32.1%) met criteria for FA symptoms, with similar prevalence among Tx (32.6%) and non-Tx (30.6%) participants despite marked differences in clinical characteristics. Across both cohorts, FA was independently associated with higher body mass index, greater psychological distress, and greater alcohol dependence severity. Childhood trauma and poorer sleep quality were additionally associated with FA among treatment-seeking participants, whereas alcohol-related measures differed according to treatment status.

**Conclusions and Relevance:**

FA was common among adults with AUD and was associated with greater psychological, behavioral, and metabolic burden regardless of treatment-seeking status. These findings suggest that FA identifies a clinically meaningful subgroup of individuals with AUD who may benefit from more comprehensive assessment and integrated treatment approaches.

**KEY POINTS BOX:**

**Question**

Are food addiction (FA) symptoms prevalent among individuals with alcohol use disorder (AUD)?

**Findings**

In this cross-sectional study of 743 adults with AUD, approximately one-third exhibited FA symptoms. Similar prevalence was observed among individuals seeking treatment for AUD and those not seeking treatment for AUD despite differences in clinical characteristics. FA symptoms were associated with higher body mass index, greater psychological distress, and greater alcohol dependence severity, with some associations differing by treatment status.

**Meaning**

FA may identify a clinically meaningful subgroup of individuals with AUD, regardless of treatment-seeking status, characterized by greater psychological and addiction-related burden, supporting further investigation of routine assessment and integrated treatment approaches.

**Abstract Figure:**

## Full Text Availability

The license terms selected by the author(s) for this preprint version do not permit archiving in PMC. The full text is available from the preprint server.

